# The Ethanol Extract of Evodiae Fructus and Its Ingredient, Rutaecarpine, Inhibit Infection of SARS-CoV-2 and Inflammatory Responses

**DOI:** 10.3390/ijms24010762

**Published:** 2023-01-01

**Authors:** Shengying Lin, Xiaoyang Wang, Hongsheng Guo, Niyu Dai, Roy Wai-Lun Tang, Hung Chun Lee, Ka Wing Leung, Tina Ting-Xia Dong, Sarah E. Webb, Andrew L. Miller, Karl Wah-Keung Tsim

**Affiliations:** 1 Center for Chinese Medicine, Division of Life Science, The Hong Kong University of Science and Technology, Clear Water Bay, Kowloon, Hong Kong, China; 2 State Key Laboratory of Molecular Neuroscience, The Hong Kong University of Science and Technology, Hong Kong, China

**Keywords:** SARS-CoV-2, natural products, traditional Chinese medicine, S-protein ELISA, 3CL protease, anti-inflammatory, cytokines

## Abstract

COVID-19, derived from SARS-CoV-2, has resulted in millions of deaths and caused unprecedented socioeconomic damage since its outbreak in 2019. Although the vaccines developed against SARS-CoV-2 provide some protection, they have unexpected side effects in some people. Furthermore, new viral mutations reduce the effectiveness of the current vaccines. Thus, there is still an urgent need to develop potent non-vaccine therapeutics against this infectious disease. We recently established a series of detecting platforms to screen a large library of Chinese medicinal herbs and phytochemicals. Here, we reveal that the ethanolic extract of Evodiae Fructus and one of its components, rutaecarpine, showed promising potency in inhibiting the activity of 3C-like (3CL) protease, blocking the entry of the pseudo-typed SARS-CoV-2 (including wild-type and omicron) into cultured cells. In addition, inflammatory responses induced by pseudo-typed SARS-CoV-2 were markedly reduced by Evodiae Fructus extract and rutaecarpine. Together our data indicate that the herbal extract of Evodiae Fructus and rutaecarpine are potent anti-SARS-CoV-2 agents, which might be considered as a treatment against COVID-19 in clinical applications.

## 1. Introduction

The COVID-19 pandemic, caused by the SARS-CoV-2 virus, has caused unprecedented damage to human health since its outbreak in 2019. As of 28 October 2022, more than 626 million confirmed cases and a death toll of over 6.5 million have been attributed to this deadly disease [1]. The original SARS-CoV-2 virus has already mutated several times, leading to several COVID-19 variants. To date, five variants of concern (VOCs) have been identified in various countries, including B.1.1.7 (UK), B.1.351 (South Africa), P.1 (Brazil), B.1.617.2 (India), and B.1.1.529 (various countries). These variants were named with Greek letters—alpha (α), beta (β), gamma (γ), delta (δ), and omicron (ο), respectively [2,3]. Each variant has distinct characteristics; for example, the omicron variant exhibits enhanced infectivity, transmissibility, and immune escape [2,3]. In addition to vaccines, there are currently several oral drugs (e.g., Paxlovid, Lagevrio, and remdesivir) that have been approved or authorized for emergency use, to help combat the disease. Nevertheless, these treatments have some drawbacks, such as side effects and limited efficacy in clinical applications [2,3]. This indicates that there is still an urgent need to develop additional potent non-vaccine-based therapeutics to combat this disease.

Several lines of evidence show that the entry of SARS-CoV-2 into host cells is initiated by the spike (S)-protein [4,5,6]. Mature S-protein consists of two non-covalently associated domains, namely S1 and S2. In the S1 domain, there is a receptor binding domain (RBD), which binds to angiotensin-converting enzyme II (ACE2) and anchors the virus to the surface of a host cell [4,5]. The S2 domain then activates cell membrane fusion via a fusion peptide, which allows the virus to enter the cell. Thereafter, viral replication and growth are mediated by a group of non-structural proteins, including 3CL protease, papain-like (PL) protease, RNA polymerase, and others [4,5]. The RNA polymerase controls the transcription and replication of viral RNA, and the 3CL and PL proteases are both key proteins in the viral replication process by regulating the host cell responses [6]. Together, these three enzymes comprise a network that drives the entry and replication of SARS-CoV-2, which results in a viral infection leading to the main common symptoms (i.e., fever, cough, lung inflammation, and excessive immune response) and ultimate somatic damage within the lungs and other organs. Thus, inhibitors against one or more of these protein targets might display anti-SARS-CoV-2 effects and lead to possible treatments for this disease [7].

Traditional Chinese medicines (TCM), which originate from natural products, have been utilised for hundreds of years to successfully combat epidemic and pandemic diseases [8]. In recent times, TCM was employed in 2003, as one of the first-line treatments in fighting SARS-CoV-1 in China. According to TCM theory, COVID-19 results from a combination of dampness, stasis, heat, and toxins. Treatments to remove any of these factors might therefore be considered for clinical application [9,10]. Indeed, several TCM prescriptions, including both Huashi Baidu Formula and Qingying decoction, have been recommended by the National Health Committee (NHC) of China. These herbal medicines are reported to remove heat from the lungs and heart, in accordance with TCM theory. Thus, TCM is a rich source of natural products for developing effective novel treatments against SARS-CoV-2 and other coronaviruses (CoVs) that arise in the future [9,10].

In order to discover possible hits from a TCM library, we recently established a series of testing platforms to screen various TCM herbal extracts/phytochemicals against viral entry of SARS-CoV-2. In previous reports, we demonstrated that extracts of Polygoni Multiflori Radix and Polygoni Cuspidati Rhizoma et Radix as well as their key chemical components, epigallocatechin gallate and gallic acid, respectively, blocked viral entry of SARS-CoV-2 [11,12]. Here, we reveal that the ethanol (EtOH) extract of Evodiae Fructus and one of its chemical components, rutaecarpine, can inhibit the activity of 3CL protease and S-protein-ACE2 interaction, and thus prevent both wild-type and omicron variants of SARS-CoV-2 from entering cultured cells. We also show that the herbal extract and its key chemical reduce the inflammatory response, as induced by SARS-CoV-2, in macrophages.

## 2. Results

### 2.1. HPLC Fingerprinting of EVF_EtOH_

**Figure 1:**
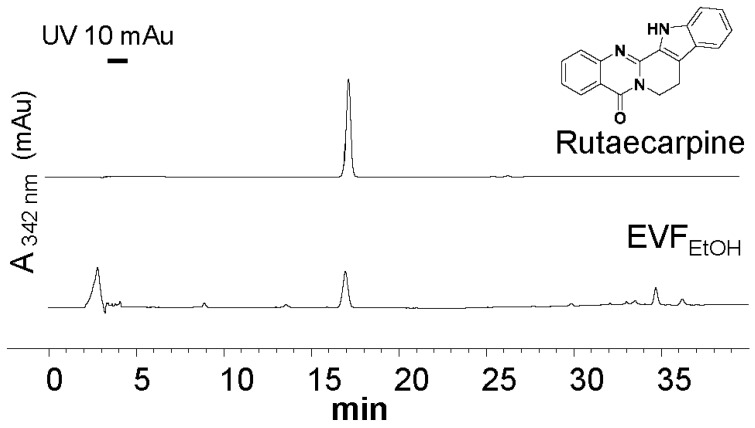
HPLC analysis of the ethanol extract of Evodiae Fructus (EVF_EtOH_). The characteristic peak of pure rutaecarpine (0.5 mg/mL; upper trace) was found in the EVF_EtOH_ extract (0.1 mg/mL; lower trace) at 10 µL. Absorbance was measured at 342 nm.

By extracting Evodiae Fructus (10 g) in EtOH, approximately 2.29 g of dry extract was obtained (herein called EVF_EtOH_) and the extraction efficiency was 22.9%. An HPLC analysis was conducted to determine the characteristic spectrum of EVF_EtOH_ and rutaecarpine was found to be one of the main components of this extract. Indeed, EVF_EtOH_ was found to contain ~0.42% rutaecarpine on a dried weight basis (Figure 1).

### 2.2. 3CL Protease and S-Protein Inhibition

**Figure 2:**
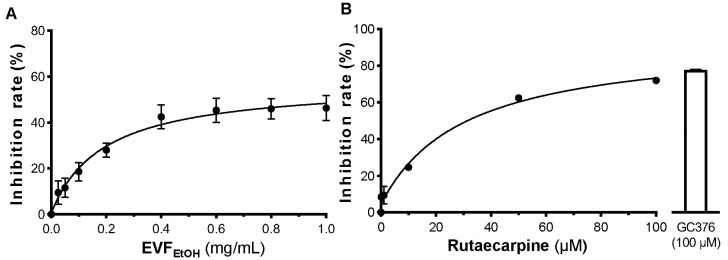
EVF_EtOH_ and rutaecarpine inhibit 3CL protease activity. EVF_EtOH_ (**A**) and rutaecarpin (**B**) both inhibited the activity of the enzyme in a dose-dependent manner. GC376 (100 µM) was used as a positive control. The data represent the mean ± SD percentage of inhibition to the control (with no drug treatment), and *n* = 4.

EVF_EtOH_ was found to inhibit the activity of 3CL protease in a dose-dependent manner from 0.01 mg/mL to 1 mg/mL, with a maximal inhibition rate of ~40% (Figure 2A). Intriguingly, a maximal inhibitory activity of ~70% was observed for rutaecarpine (Figure 2B). This is therefore almost as potent as the positive control, GC376 (a well-known inhibitor of 3CL protease), which inhibited the activity of this enzyme by ~75% inhibition. These results indicate that EVF_EtOH_ and its main chemical component, rutaecarpine, can disrupt 3CL protease activity and therefore can potentially block the viral replication and growth of SARS-CoV-2.

**Figure 3:**
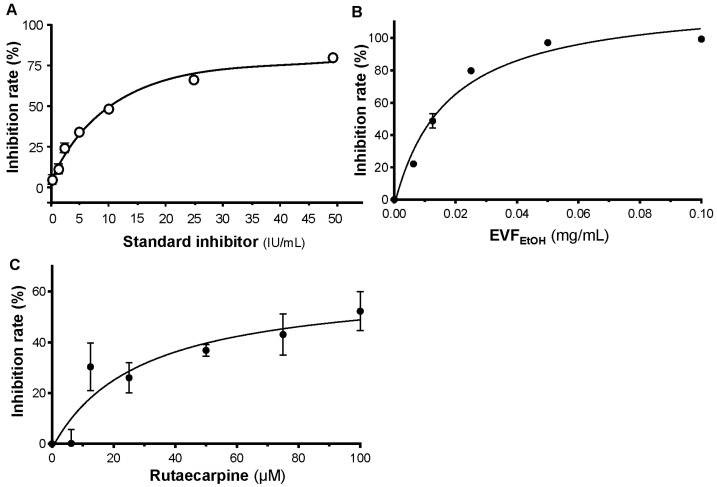
EVF_EtOH_ and rutaecarpine inhibit the binding of S-protein to ACE2. (**A**) A standard inhibitor (calibrated to NIBSC code 20/136) was utilised as a positive control. EVF_EtOH_ (**B**) and rutaecarpine (**C**) both disrupted the binding between the S-protein and the ACE2 receptor. The data represent the mean ± SD percentage of inhibition to control (with no drug treatment), and *n* = 4.

In addition, an S-protein-based ELISA was employed to investigate if EVF_EtOH_ blocked the interaction between S-protein and ACE2. A standard inhibitor (NIBSC code 20/136) was utilised as a positive control (Figure 3A). Our data showed that EVF_EtOH_ inhibited the S-protein-ACE2 interaction in a dose-dependent manner with an IC_50_ of ~0.01 mg/mL and a maximal inhibition of ~100% was achieved at 0.05 mg/mL (Figure 3B). We also showed that rutaecarpine attenuated S-protein-ACE2 binding in a dose-dependent manner with an IC_50_ of ~30 µM (Figure 3C). These data indicate that rutaecarpine might be (at least in part) responsible for disrupting the interaction between S-protein and ACE2 receptor by EVF_EtOH_.

### 2.3. EVF_EtOH_ and Rutaecarpine Block Viral Entry of Pseudotyped SARS-CoV-2

**Figure 4:**
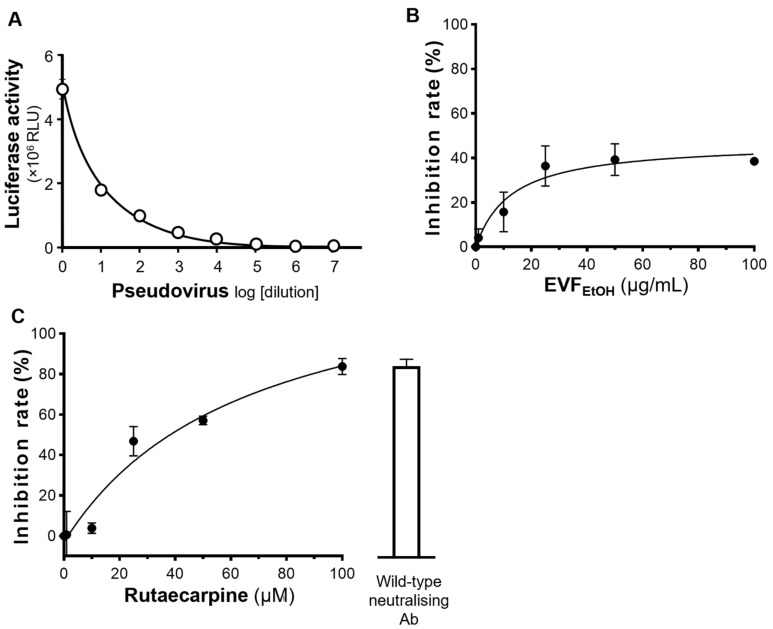
EVF_EtOH_ and rutaecarpine block entry of wild-type pseudovirus into HEK293T cells overexpressing ACE2. (**A**) The ELISA assay successfully demonstrated the entry of pseudovirus into these cells, exhibiting a dose-dependent response to dilution of the virus. (**B**) EVF_EtOH_ and (**C**) rutaecarpine blocked entry of the pseudovirus into the cells in dose-dependent manner. SARS-CoV-2 neutralising antibody was used at 1 µg/mL as a positive control. The values represent the mean ± SD percentage of inhibition to control (with no drug treatment) and *n* = 3.

A pseudovirus entry platform was established with cultured HEK293T cells overexpressing ACE2 receptors on their plasma membrane. These cells were treated with a pseudotyped SARS-CoV-2 containing S-protein and luciferase. The relative luciferase activity (revealed by the amount of luminescence generated) indicated the level of pseudovirus inside the host cells (Figure 4A). Thus, drugs that inhibit pseudotyped SARS-CoV-2 entry into cells are expected to reduce luciferase activity. First, to test if EVF_EtOH_ and rutaecarpine are toxic to the cells, an MTT (3-(4,5-dimethylthiazol-2-yl)-2,5-diphenyltetrazolium bromide) assay was conducted. As anticipated, the cell viability remained high in the presence of either EVF_EtOH_ or rutaecarpine, at concentrations up to 100 µg/mL and 100 µM, respectively (Figure S1). Thus, both EVF_EtOH_ and rutaecarpine are unlikely to cause cell death at the applied doses.

**Figure 5:**
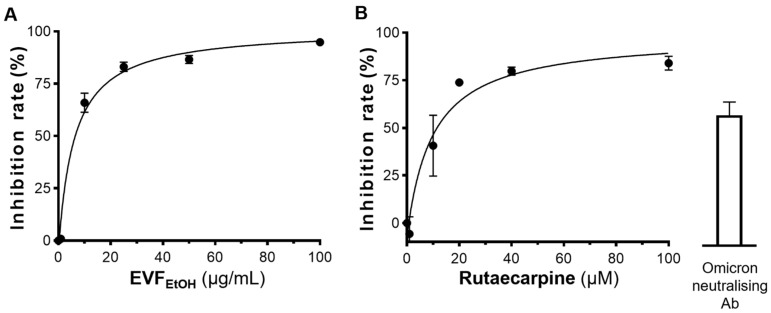
EVF_EtOH_ and rutaecarpine block entry of the omicron pseudovirus into HEK293T cells overexpressing ACE2. (**A**) EVF_EtOH_ and (**B**) rutaecarpine both blocked omicron pseudovirus entry in dose-response manners. SARS-CoV-2 neutralising antibody was used at 1 µg/mL, as a positive control. The data represent the mean ± SD percentage of inhibition to control (with no drug treatment) and *n* = 3.

Having tested the ELISA assay with decreasing dilutions of the wild-type SARS-CoV-2 pseudovirus (Figure 4A), we next determined if EVF_EtOH_ could block entry of the pseudovirus into the ACE-2 overexpressing cells. Our data showed that, indeed, EVF_EtOH_ inhibited pseudovirus entry in a dose-response manner from 0.1 µg/mL to 100 µg/mL (Figure 4B). In addition, rutaecarpine attenuated viral entry with a maximum inhibition rate of ~80%, which is as high as that of the neutralising antibody positive control (Figure 4C). We subsequently showed that EVF_EtOH_ and rutaecarpine also displayed robust inhibitory activity against host cell entry of an omicron SARS-CoV-2 pseudovirus in a dose-dependent manner. Specifically, EVF_EtOH_ was effective from 10 µg/mL to 100 µg/mL, whereas rutaecarpine showed an even higher potency than the neutralising antibody at ~15 µM (Figure 5A,B). Indeed, EVF_EtOH_ and rutaecarpine both displayed better inhibitory performance against the omicron variant than that against wild-type SARS-CoV-2.

**Figure 6:**
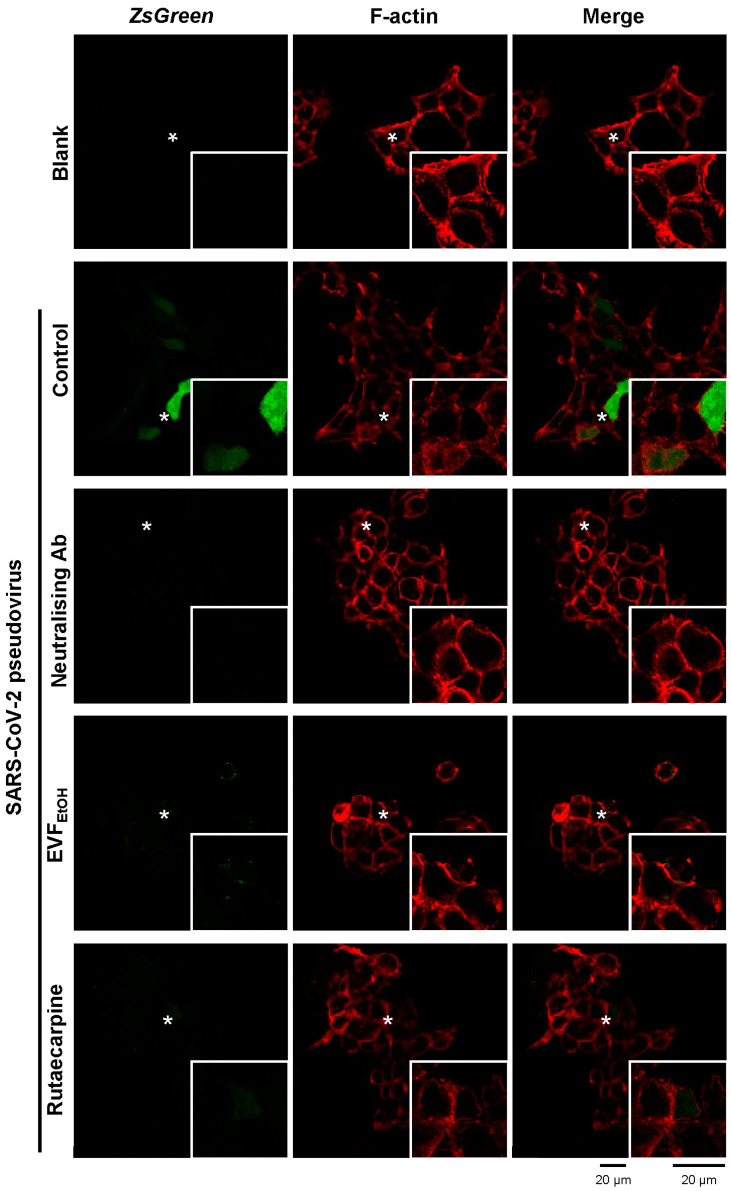
Representative confocal images showing that EVF_EtOH_ and rutaecarpine prevent pseudovirus entry into cultured HEK293T cells. The pseudovirus expressed ZsGreen which generated green fluorescence (color green in the figure) inside cells, and fluorescent phalloidin (color red in the figure) was used to stain the F-actin component of the cell membrane. Prior to exposure to the pseudovirus, cells were either untreated (control) or were treated with a SARS-CoV-2 neutralising antibody (1 µg/mL; positive control), or with EVF_EtOH_ (0.1 mg/mL) or rutaecarpine (100 µM). The fluorescence intensity was significantly decreased following treatment with EVF_EtOH_ or rutaecarpine compared with the untreated controls. In each panel, the region indicated by the asterisk is shown at higher magnification in the inset image. For each treatment *n* = 3.

We also employed an immunofluorescence assay to investigate the inhibitory effects of EVF_EtOH_ and rutaecarpine on pseudoviral entry. In this assay, ZsGreen expressed by the pseudovirus generated green fluorescence intracellularly when the host cells were infected. As shown in Figure 6, control cultures showed increased green fluorescence intracellularly, following infection with the wild-type SARS-CoV-2 pseudovirus. However, the amount of GFP was markedly reduced following treatment with EVF_EtOH_ or rutaecarpine. This is similar to the decreased fluorescence observed with the neutralising antibody (positive control).

**Figure 7:**
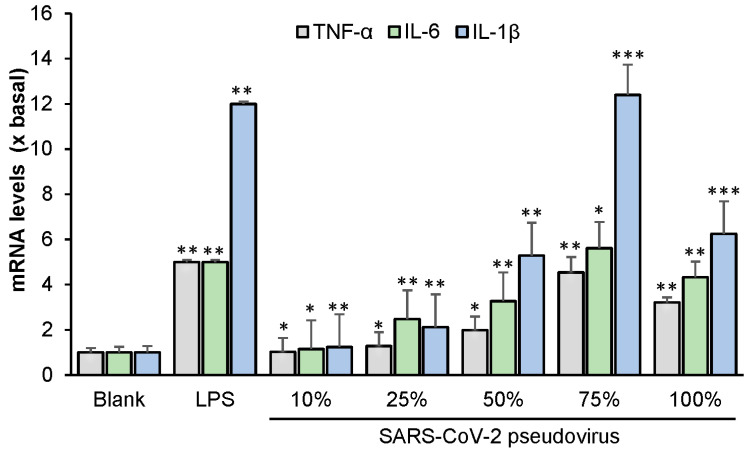
SARS-CoV-2 induces the expression of inflammatory cytokines in cultured macrophages. The mRNA levels of TNF-α, IL-6, and IL-1β were measured by RT-PCR. The data indicate the mean ± SD (*n* = 5) fold change against the blank group (x basal), and the asterisks indicate statistically significant differences such that * *p* <0.05, ** *p* < 0.01, *** *p* < 0.001 compared with the blank group.

Several lines of evidence indicate that the inflammatory response is a common symptom of COVID-19 among patients [13,14]. TNF-α, IL-6 and IL-1β are recognized as being crucial cytokines in the inflammatory response. Therefore, here we measured the mRNA levels of TNF-α, IL-6 and IL-1β in RAW 264.7 cells infected with wild-type SARS-CoV-2. LPS (used as a positive control) markedly increased the levels of TNF-α, IL-6 and IL-1β relative to the blank (Figure 7). Various concentrations of the virus were then applied, of which a 75% diluted concentration resulted in the highest level of cytokine mRNA. Cell apoptosis was observed at a viral concentration of 100%; as expected, this led to a reduction in the level of each cytokine.

### 2.4. EVF_EtOH_ and Rutaecarpine Reduce the Inflammatory Response Induced by SARS-CoV-2

**Figure 8:**
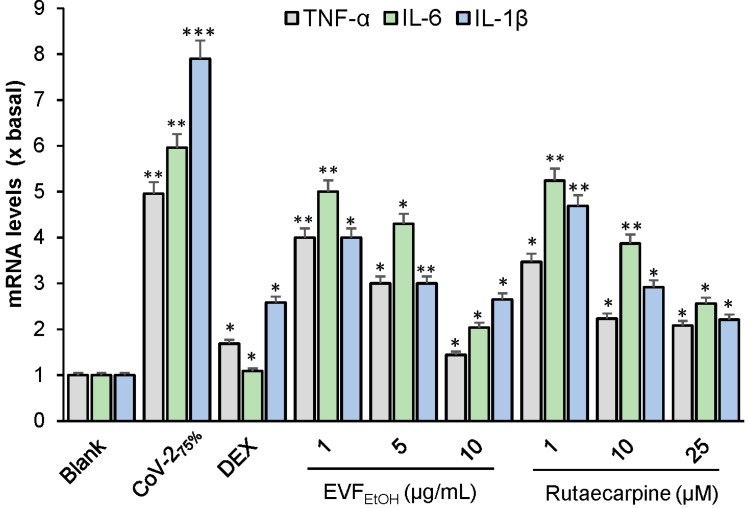
EVF_EtOH_ and rutaecarpine suppress SARS-CoV-2-induced inflammation in cultured macrophages. The mRNA levels of the inflammatory cytokines TNF-α, IL-6, and IL-1β, were measured by RT-PCR. The data indicate the mean ± SD (*n* = 5) fold change against the blank group (x basal), and the asterisks indicate statistically significant differences such that * *p* < 0.05, ** *p* < 0.01, *** *p* < 0.001 compared with the blank group.

Next, we evaluated the effect of EVF_EtOH_ and rutaecarpine on the SARS-CoV-2 stimulated increase in mRNA levels of the inflammatory cytokines. Dexamethasone (a well-known anti-inflammatory drug) served as a positive control; this displayed up to 80% inhibition of the mRNA levels of TNF-α, IL-6 and IL-1β (Figure 8). EVF_EtOH_ and rutaecarpine also significantly reduced the levels of SARS-CoV-2-induced cytokines in a dose-dependent manner from 1 µg/mL to 10 µg/mL and 1 µM to 25 µM, respectively (Figure 8). These results suggest that EVF_EtOH_ might be useful in attenuating inflammation in COVID-19 patients and that the rutaecarpine component of this herbal extract is responsible for its anti-inflammatory effects. To further validate our observations, several docking studies were conducted to measure the affinities between rutaecarpine and various targeted proteins. As shown in Figure S2, rutaecarpine binds to the S-protein of wild-type and omicron SARS-CoV-2 with energies of −8.6 KJ/mol and −10.2 KJ/mol, respectively. These docking investigations support our in vitro studies by indicating that rutaecarpine can block viral entry of SARS-CoV-2.

## 3. Discussion

TCM has a long and very successful history in combating various diseases, including viral epidemics. Interestingly, several TCM prescriptions and therapeutics have already been reported to attenuate fever among COVID-19 patients. These include Jinhua Qinggan Granule and Lianhua Qingwen Capsule [15]. Evodiae Fructus is the dried fruit of an herb called *Evodia rutaecarpa* (Juss.) Benth. and it has been utilised by TCM practitioners for thousands of years due to its potent anti-obesity, anti-cancer, and anti-inflammatory effectiveness in clinical applications [16]. In addition, Chiou et al. [17] reported that Evodiae Fructus displays promising efficacy against the H1N1 influenza A virus. This inspired us to investigate if Evodiae Fructus might also exert antiviral activity against COVID-19. The data were acquired from our screening platform and indicated that the EtOH extract of Evodiae Fructus does indeed show robust inhibition to pseudotyped SARS-CoV-2 by targeting various key proteins in the viral entry and infection pathway, such as 3CL and the S-protein-ACE2 complex. This also suggested that Evodiae Fructus and its components are likely to express antiviral activity to the live full-length SARS-CoV-2.

Various other phytochemicals have been shown to play important roles in targeting the SARS-CoV-2 protein network, thereby limiting viral entry and replication [18]. For example, herbacetin, apigenin-7-O-rhamnoglucoside, and pectolinarin are all potent inhibitors of 3CL protease, blocking viral replication at a concentration of 20 µM [18,19]. In addition, theaflavin (found in black tea) was shown to exhibit anti-SARS-CoV-2 effects by blocking RNA-dependent RNA polymerase, and hesperidin robustly inhibits S-protein-ACE2 interaction and thus blocks viral entry into host cells. Various alkaloids have also been shown to have significant inhibitory effects on the activity of various coronaviruses. For example, lycorine, tetrandrine, fangchinoline, and cepharanthine are all reported to be effective inhibitors of SARS-CoV-2, with relatively low IC_50_ values of 15.7 nM, 0.33 µM, 1.01 µM, and 0.83 µM, respectively [18,20]. As an alkaloid-type phytochemical, rutaecarpine has previously been reported to inhibit cyclooxygenase-2 and acetylcholinesterase (AChE) [21]. Here, we reveal that rutaecarpine can inhibit 3CL protease and block the binding of S-protein to ACE2, and so in this way it disrupts viral entry and replication. It is worth mentioning that some components within EVF_EtOH_, such as evodine, evodiamine, and evocarpine, could potentially express antiviral effects and contribute to the anti-SARS-CoV-2 efficacy of EVF_EtOH_ [16]. As such, these phytochemicals could be interesting targets for future screening.

We also showed that the SARS-CoV-2 pseudovirus induces an inflammatory response in RAW264.7 cells. This supports findings reported by Khan et al. [22] and Cao et al. [23] who previously demonstrated that the SARS-CoV-2 S-protein induces inflammatory responses in various cell lines, including A549, RAW264.7, and THP-1 cells. In these previous reports, it was generally believed that the inflammatory response was triggered by TLR2 and activation of the NF-κB pathway [22,23]. Here, we demonstrated that EVF_EtOH_ and rutaecarpine could reduce the inflammatory response, as induced by the SARS-CoV-2 pseudovirus.

We were especially interested in the anti-SARS-CoV-2 effects of rutaecarpine as its IC_50_ values against 3CL protease and S-protein binding are relatively high when compared with other reported inhibitors. This suggests that rutaecarpine is a potential lead candidate for fragment-based structural modification in optimising its efficacy in future investigations. Interestingly, our docking studies revealed that rutaecarpine binds to the same site in S-protein for both wild-type and omicron SARS-CoV-2 (Figure S2). The indole scaffold (highlighted in blue) of this ligand displays good affinity to Phe490 via pi-pi stacking interaction, indicating that this fragment might be retained in the subsequent structural optimization. The docking images indicate that the C-2 position requires high energy at the binding site, so several chemical vectors could be introduced at this position to improve the binding affinity. Given the fact that Tyr449 has been found around the C-2 position, we suggest that chemical substituents, such as amine, amide, and other carbonyl groups, might establish hydrogen bonds with this amino acid, allowing the ligand to be well accommodated at the binding site and thus reduce the binding energy.

## 4. Materials and Methods

### 4.1. Cell Culture

HEK293T cells and RAW264.7 cells (American Type Culture Collection, Manassas, VA, USA) were maintained in high glucose Dulbecco’s Modified Eagle Medium (DMEM) supplemented with 1% penicillin/streptomycin and 10% fetal bovine serum (Thermo Fisher Scientific, Waltham, MA, USA; herein called culture medium) at 37 °C in an incubator with water-saturated atmosphere and 5% CO_2_. Fresh culture medium was supplied every other day. HEK293T cells overexpressing human ACE2 (hACE2) were prepared by transfection with the pcDNA3.1-hACE2 plasmid (Addgene, Watertown, MA, USA). The cell viability was determined, as described previously [24], except absorbance was measured at 570 nm.

### 4.2. Preparation of Herbal Extracts

Evodiae Fructus (the dried, ripe fruit of *Evodia rutaecarpa* Hort.) was purchased from local herbal market and authenticated in accordance with the Hong Kong Chinese Materia Medica Standards [25]. Evodiae Fructus powder (10 g) was placed in a 250 mL round-bottomed flask and dissolved in 100 mL distilled 90% ethanol to obtain ethanol extract. The solution was then refluxed for 1 h, after which it was filtered through a paper filter (110 µm, Advantec, Tokyo, Japan). The extract was then evaporated to dryness with a rotary evaporator, providing a yield of 2.29 g ethanol extract (EVF_EtOH_), and the extraction efficiency was, therefore, determined to be 22.9%.

### 4.3. HPLC Analysis of EVF_EtOH_ Extract

HPLC was conducted according to methods developed by the Hong Kong Chinese Materia Medica Standards [25]. EVF_EtOH_ (1 mg) was placed in a conical flask and dissolved in 10 mL EtOH (50%). Rutaecarpine (at purity >95% from Chengdu Must, Chengdu, China) was used as an HPLC standard and prepared at 500 mg/L in 100% EtOH. Each solution was sonicated for 30 min and then filtered through a polytetrafluoroethylene membrane syringe filter (0.45 µm; Anpel Laboratory Technologies, Shanghai, China), after which the solution was transferred to a 10 mL volumetric flask filled with 50% EtOH. For HPLC, the mobile phase consisted of Milli Q water and acetonitrile (ACN) using a gradient condition as follows: 0–20 min, 55% ACN; 20–30 min, 55–100% ACN; and 30–40 min, 100% ACN. The flow rate of the mobile phase was 1.0 mL/min, and the injection volume was 10 μL. The characteristic peaks were detected at wavelength of 342 nm.

### 4.4. Production of SARS-CoV-2 Pseudotyped Virus

HEK293T cells at 80% confluence were transfected with various components of the SARS-related coronavirus 2, Wuhan-Hu-1 Spike-pseudotyped Lentiviral Kit (NR-52948; BEI Resources, National Institute of Allergy and Infectious Diseases, Rockville, MD, USA), including the SARS-CoV-2 spike glycoprotein (NR-52514 for the wild-type or 179907 for the omicron variant), a lentiviral backbone expressing gene Luciferase and ZsGreen (NR-52516), and several helper plasmids (NR-52517, NR-52518, and NR-52519) using Lipofectamine™ 3000 (Thermo Fisher Scientific) or JetPRIME (Poly-Plus, Shanghai, China) transfection reagent, following the manufacturer’s instructions. After 72 h, SARS-CoV-2 pseudotyped-virus (defined as pseudovirus) particles were collected and passed through a 0.45 µm filter (Sartorius, Goettingen, Germany) before being used directly in the subsequent experiments.

### 4.5. Inhibiting SARS-CoV-2 Pseudovirus Entry

The ACE2-overexpressing HEK293T cells were seeded into 48-well plates and incubated with 400 µL culture medium containing SARS-CoV-2 pseudovirus (100 µL) plus EVF_EtOH_ or rutaecarpine at 37 °C for 24 h. This medium was then replaced with fresh culture medium, and the cultures were allowed to recover for 48 h, after which they were washed with PBS and the amount of luciferase was quantified. EVF_EtOH_ was tested at final concentrations of 0.1, 1, 10, 25, 50, and 100 µg/mL, and rutaecarpine was tested at concentrations of 1, 10, 25, 50 and 100 µM. An anti-SARS-CoV-2 neutralising antibody (A19215, ABClonal, Woburn, MA, USA) was used as a positive control (1 µg/mL), whereas a solvent blank (without the pseudovirus) was used as a negative control. The percentage inhibition of EVF_EtOH_ or rutaecarpine was determined by the luciferase activity normalized when compared with that without any treatments. The luciferase assay was conducted, as previously described [24]. The percentage inhibition of each sample was calculated as follows: Inhibition rate = (Luciferase activity of the solvent blank − Luciferase activity of the sample)/(Luciferase activity of the solvent blank − Luciferase activity of group without pseudovirus) × 100%.

### 4.6. Fluorescence Labelling

Cultured cells treated with the SARS-CoV-2 pseudovirus (expressing ZsGreen) and EVF_EtOH_ or rutaecarpine as described above, were fixed with 4% paraformaldehyde for 30 min. They were then incubated with 5% BSA for 1 h. The F-actin component of the cell membrane was then stained with fluorescent rhodamine phalloidin (Thermo Fisher Scientific, Waltham, MA, USA). Images were acquired with a confocal inverted laser microscope (Leica SP8) with 63× magnification at excitation wavelengths of 488 nm for Zsgreen, and 555 nm for F-actin/phalloidin.

### 4.7. Screening of S-Protein Inhibitor

S-protein inhibition was analysed with the SARS-CoV-2 Spike-ACE2 binding assay kit (ImmunoDiagnostics Ltd. Hong Kong, China) according to the manufacturer’s instructions. EVF_EtOH_ was tested at concentrations of 0.00625, 0.0125, 0.025, 0.05, and 0.1 mg/mL, and rutaecarpine was tested at concentrations of 6.25, 12.5, 25, 50, 75, and 100 µM. Reactions were terminated via the addition of 2 M H_2_SO_4_, and data were acquired with a microplate reader (FlexStation; Molecular Devices, San Jose, CA, USA). The percentage of inhibition was calculated as follows: Percentage of inhibition = (P_Avg_ − S_Avg_)/P_Avg_ × 100%, where P_Avg_ and S_Avg_ were the mean OD values of the positive control and test samples, respectively.

### 4.8. Screening 3CL Protease Inhibition

Samples were tested for their ability to bind 3CL protease on a fluorogenic substrate with the SensoLyte SARS-CoV-2 3CL protease assay kit (AnaSpec, San Jose, CA, USA) according to the manufacturer’s instructions. When 3CL protease was bound with the substrate, fluorescence at 460 nm was generated following excitation at 360 nm. EVF_EtOH_ was tested at concentrations of 0.025, 0.05, 0.1, 0.2, 0.4, 0.6, 0.8, and 1 mg/mL, whereas rutaecarpine was tested at concentrations of 0.1, 1, 10, 50, and 100 µM. The percentage of inhibition was calculated as: percentage of inhibition = (P_Avg,b_ − S_Avg_, b)/P_Avg,b_ × 100%; where P_Avg_,_b_ and S_Avg_,_b_ were the mean fluorescence of positive control and test samples, respectively, subtracted from the mean fluorescence of the blank.

### 4.9. Testing the Inhibition of Inflammation

RAW264.7 cells were seeded in 12-well plates at 10^6^/mL and incubated overnight. Wild-type SARS-CoV-2 virus was then added to the medium at concentrations of 100%, 75%, 50%, 25%, 10%, and 0% (*v*/*v*) for 24 h. LPS (0.1 µg/mL) was employed as a positive control. In another series of experiments, RAW264.7 cells incubated with a diluted concentration of 75% wild-type SARS-CoV-2 virus were treated with EVF_EtOH_ (at concentrations of 1, 5, or 10 µg/mL) or rutaecarpine (at concentrations of 1, 10, or 25 µM) for 24 h prior to RNA extraction and RT-PCR. Dexamethasone at concentration of 10 µM was used as a positive control. The mRNA levels of TNF-α, IL-6, and IL-1β were measured by RT-PCR.

### 4.10. RNA Extraction and RT-PCR

RNAzol@RT reagent (Molecular Research Center, Cincinnati, OH, USA) was used to extract total RNA from RAW264.7 cells. The cells were incubated in RNAzol@RT at room temperature. The total RNA was precipitated in 75% ethanol (*v*/*v*) by centrifugation at 12,000× *g* for 10 min. The RNA pellet was then washed with 75% ethanol and dissolved in RNAase-free water. The RNA quality was determined by NanoDrop™ (Thermo Fisher Scientific) based on the ratio of absorbance at 260–280 nm (~2.0). One μg of RNA per sample was used for reverse transcription using the First Strand cDNA synthesis kit (Thermo Fisher Scientific) according to the manufacturer’s protocol. Forty-five cycles of amplification were performed, and each cycle consisted of denaturation at 95 °C for 30 s, annealing at 55 °C for 30 s, and extension at 72 °C for 20 s on a Roche Lightcycler 480 System (Roche, Basel, Switzerland). The primers used are as follows: TNF-α: AGT GAC AAG CCT GTA GCC, IL-6: TTC CAT CCA GTT GCC TTC TTG G, and IL-1β: ATG GCA GAA GTA CCT AAG CTC GC.

### 4.11. Computational Docking Analysis

The chemical structures of the phytochemicals were downloaded from Pubchem (https://pubchem.ncbi.nlm.nih.gov/, accessed on 1 September 2022), and the S-protein structure was downloaded from the Protein Data Bank (https://www.rcsb.org/, accessed on 1 September 2022). Virtual screening was performed with SEESAR (Ver. 12.0; https://www.biosolveit.de/, accessed on 1 September 2022) as follows: (i) the binding site was determined according to the residues forming the identified druggable pocket. Ligand binding states, including protonation and tautomeric forms, were subsequently evaluated using the ProToss method to generate the most accessible hydrogen network. (ii) Docking modulation was performed using the “Compute LeadIT Docking” mode in the FlexX algorithm; ten binding conformations for each ligand were generated. (iii) The binding energy (i.e., ∆G) and estimated HYDE affinity (KiHYDE) for each ligand pose were calculated using the “Assess Affinity with HYDE in SEESAR” mode in the HYDE rescoring function [26]. In the docking images, green indicates that the chemical atom is favourable at the binding pocket, whereas red indicates that the atom requires high energy to approach the binding site and is therefore not favourable.

## 5. Conclusions

In this report, we have revealed that EVF_EtOH_ can inhibit 3CL protease activity, disrupt the formation of the S-protein-ACE2 complex, prevent SARS-CoV-2 entering to HEK293T cells, and attenuate the inflammatory response, as induced by the virus. Furthermore, we show that rutaecarpine, a component of EVF_EtOH_, is responsible for at least some of the recorded efficacy of the parental TCM herb. Taken together, our data indicate that we have successfully identified EVF_EtOH_ and rutaecarpine containing both anti-SARS-CoV-2 and anti-inflammation activities. Thus, Evodiae Fructus may be considered a promising anti-COVID-19 treatment for clinical applications.

## Data Availability

The data presented in this study are available on request from the corresponding author. The data are not publicly available due to the confidentiality of this research.

## Supplementary Materials

The following supporting information can be downloaded at: https://www.mdpi.com/article/10.3390/ijms24010762/s1.
