# Supplementary material for: The Ethanol Extract of Evodiae Fructus and Its Ingredient, Rutaecarpine, Inhibit Infection of SARS-CoV-2 and Inflammatory Responses
Source: Int J Mol Sci. 2023 Jan 1;24(1):762. doi: 10.3390/ijms24010762 (PMC9821500; doi:10.3390/ijms24010762)
Supplement: Supplementary file 1 [file ijms-24-00762-s001.zip › ijms-2044619-supplementary.pdf]

The Ethanol Extract of Evodiae Fructus and Its Ingredient, Rutaecarpine, Inhibit Infection of SARS-CoV-2 and Inflammatory Responses

Shengying Lin [1,2], Xiaoyang Wang [1,2], Hongsheng Guo [1,2], Niyu Dai [1,2], Roy Wai-Lun Tang [1,2], Hung Chun Lee [1,2], Ka Wing Leung [1,2], Tina Ting-Xia Dong [1,2], Sarah E. Webb [2], Andrew L. Miller [2] and Karl Wah-Keung Tsim [1,2,*]

[1] Center for Chinese Medicine, Division of Life Science, The Hong Kong University of Science and Technology, Clear Water Bay, Kowloon, Hong Kong, China

[2] State Key Laboratory of Molecular Neuroscience, The Hong Kong University of Science and Technology, Hong Kong, China

Correspondence

Prof. Karl Wah-Keung Tsim, Center for Chinese Medicine, Division of Life Science, The Hong Kong University of Science and Technology, Clear Water Bay, Hong Kong, China

Tel.: +852-2358-7332

Fax: +852-2358-1552

Email: botsim@ust.hk

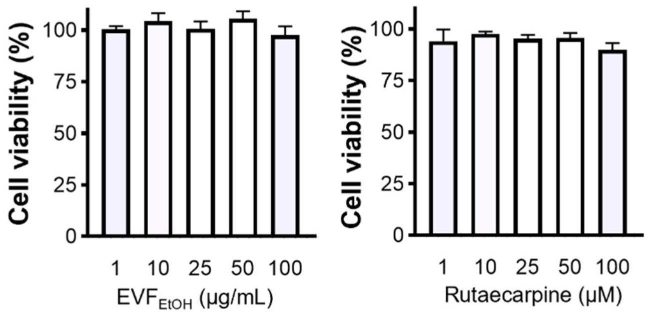

Figure S1: **Bar charts showing the viability of cells following treatment with EVF_{EtOH} or rutaecarpine.** EVF_{EtOH} was tested at final concentrations of 1, 10, 25, 50, and 100 μg/mL, whereas rutaecarpine was tested at final concentrations of 1, 10, 25, 50 and 100 μM. MTT solution was applied to give a final concentration of 0.5 mg/mL, after which the optical density was determined at 570 nm. The data show mean ± SD percentage to control (with no drug treatments) and *n* = 4.

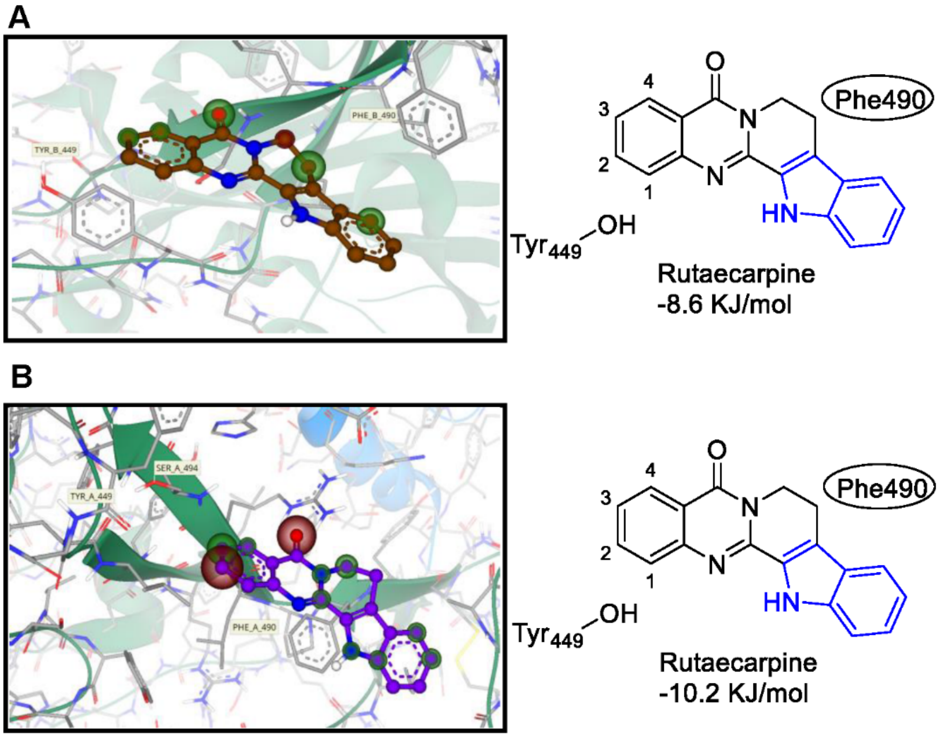

Figure S2: Docking study of rutaecarpine against the S-protein of wild-type and omicron SARS-CoV-2. (A) The receptor binding domain (RBD, residues 438-506) in the wild-type S-protein (PDB code: 6LZG) was determined to be the binding site. The chemical structures were downloaded from PubChem (https://pubchem.ncbi.nlm.nih.gov/). **(B)** The RBD (residues 438-506) of the omicron S-protein (PBD code: 7T9L) was determined to be the binding site. The binding energies are indicated in KJ/mol.
